# Nursing Interventions in Primary Care for the Management of Maladaptive Grief: A Scoping Review

**DOI:** 10.3390/nursrep14030178

**Published:** 2024-09-14

**Authors:** Martín Rodríguez-Álvaro, Pedro Ruymán Brito-Brito, Alfonso Miguel García-Hernández, Irayma Galdona-Luis, Claudio Alberto Rodríguez-Suárez

**Affiliations:** 1 Health Services Management Board of La Palma, The Canary Islands Health Service, 38713 Breña Alta, Spain; mrodrigu@ull.edu.es; 2 Nursing Department, Faculty of Healthcare Sciences, University of La Laguna, 38200 Santa Cruz de Tenerife, Spain; 3 Primary Care Management Board of Tenerife, The Canary Islands Health Service, 38004 Santa Cruz de Tenerife, Spain; igallui@gobiernodecanarias.org; 4 Research Support Unit, Insular Maternal and Child University Hospital Complex, Canary Health Service, 35016 Las Palmas de Gran Canaria, Spain; crodsuag@gobiernodecanarias.org; 5 Nursing Department, Faculty of Healthcare Science, University of Las Palmas de Gran Canaria (ULPGC), 35016 Las Palmas de Gran Canaria, Spain

**Keywords:** prolonged grief disorder, bereavement, grief, primary care nursing, community health nursing

## Abstract

Grief is a natural and self-limited adaptation process to a new reality that emerges after a significant loss (whether real or perceived), with a broad variety of manifestations that exert an impact on a grieving person’s health. The study aim was to synthesize the evidence available about the interventions carried out by Primary Health Care nurses, by means of an individual approach to reduce maladaptive grief or maladaptive grief risk. A scoping review was conducted (November and December 2023) through searches in Medline, Cinahl, Web of Science, ProQuest and Scopus using MeSH terms combined with Booleans. Primary research of any design in adult people undergoing grief situations and receiving professional assistance by nurses in the primary, home and community care contexts published after 2009 in English, Spanish or Portuguese languages were included. Excluded publications were those conducted in in-hospital clinical settings and which did not correspond to research designs or the gray literature. The screening process was carried out by two reviewers using the appropriate JBI critical appraisal tools for each design and discrepancies were resolved by a third reviewer. A total of *n* = 10 studies were included (*n* = 4 qualitative, *n* = 2 RCTs, *n* = 1 quasi-experimental, *n* = 2 cross-sectional observational, and *n* = 2 mixed methods). The qualitative studies identified topics and subtopics of professionals’ and families’ experiences of grief. The observational studies analyzed symptoms and factors associated with the grieving process. Interventions consisted of cognitive–behavioral therapies delivered by psychological specialists who assessed the severity of grief in a range of cultural contexts using different instruments. The evidence retrieved from the studies that address the reduction in maladaptive grief or maladaptive grief risk is not conclusive. There is a need to increase both the number and the methodological quality of studies assessing the effectiveness of Nursing care in Primary Health Care for individuals experiencing maladaptive grief or maladaptive grief risk. Further research should focus on experimental studies, developing specific interventions conducted by nurses to address individual’s grief and prevent maladaptive grief.

## 1. Introduction

Grief is a natural and self-limited adaptation process to a new reality that emerges after a significant loss (whether real or perceived), with a broad myriad of manifestations that exert an impact on a grieving person’s health. This impact on health produced by the loss is widely documented and implies further care needs in grieving people, increasing the associated comorbidity and the risk of death [1].

There is scarcity of studies carried out in the family and community contexts that show the impact of a Nursing intervention on the maladaptive grief process. There is limited evidence on what works best to approach types of grief (maladaptive, pathological or risk of), as well as on how to implement the intervention and to whom it should be targeted [2]. Nevertheless, the support provided by community nurses can reduce the chances of developing complications, and Nursing follow-up visits are important for this purpose [3,4].

Given the lack of methodologically reliable research studies showing the efficacy of interventions, the recommendation is that, in uncomplicated grief, the professionals’ role should be to provide information and activate the available resources, without implementing a formal or structured approach [5]. Preventive interventions seem not to be effective, but follow-up ones do, as they can reduce complicated grief symptoms [6]. In a review about interventions for supporting informal caregivers in the terminal phase of a disease, Candy et al. show that the support interventions for caregivers do not significantly reduce short-term psychological disorders or improve their coping skills or quality of life [7].

In risk grief, it is advisable to implement periodic follow-up instances, providing emotional support and valuing individual needs. Regarding uncomplicated grief, the review by Currier et al. finds that interventions targeted exclusively at patients with major difficulties adapting to the loss do yield beneficial results [8].

The systematic review by Nagraj and Barclay [9] about grief care in Primary Health Care (PHC) identifies considerable variability, both in the type of visit and in the grieving process follow-up. Despite the scarce evidence available, it is estimated that it would be appropriate for family physicians and nurses to offer support to grieving people in a productive, receptive and non-intrusive way [9]. The usual contact after a loss consists of a home visit, especially if there was a previous relationship [10]. It is known that a grieving person’s satisfaction increases if the health team has helped them undergo a peaceful grieving process [11].

The following research question was our starting point: Which interventions nurses implement in the PHC context by means of an individual approach to reduce maladaptive grief or maladaptive grief risk in adults? The aim was to synthesize the evidence available about the interventions carried out by PHC nurses, by means of an individual approach to reduce maladaptive grief or maladaptive grief risk.

## 2. Materials and Methods

Design: A scoping review has been developed, following the methodology proposed by the Joanna Briggs Institute (JBI) [12]. The criteria set forth in the Preferred Reporting Items for Systematic Reviews and Meta-Analyses extension for Scoping Reviews (PRISMA-ScR) [13] and in Enhancing Transparency in Reporting the Synthesis of Qualitative Research (ENTREQ) [14] were followed to report the results. The research question has followed the structure comprising Problem (P): People undergoing maladaptive grief or at risk of maladaptive grief; Phenomenon of interest (I): Individual Nursing interventions in the PHC context; and Outcome (O): Grief process resolution. The review protocol has been registered and is available in https://osf.io/gawjr/ (accessed on 12 February 2023) (DOI: 10.17605/OSF.IO/GAWJR).

Information sources: As a first step, the scientific literature was consulted to determine the existence or not of systematic reviews addressing the topic and a search was conducted in PROSPERO to find registered protocols corresponding to surveys that answered the same research question. After this initial query, searches were conducted in the following databases: Medline (through PubMed and OvidSP), Cinahl (through EbscoHOST), Web of Science (WOS) (through WOS Complete), ProQuest (through ProQuest Complete) and Scopus (through Scopus-Elsevier). The TripDatabase search engine was consulted to complete the process.

Search strategies: The searches were conducted in November and December 2023, defining a time limit to records published after 2009; this criterion was chosen for being the year when the searches for the review by Nagraj and Barclay [9] were conducted. The search strategies included the following MeSH descriptors in title and abstracts: “Prolonged Grief Disorder” (PGD) OR “Bereavement” OR “Grief” OR “Disenfranchised Grief” AND “Primary Care Nursing” OR “Community Health Nursing” OR “Home Health Nursing” OR “Nurses, Community Health” OR “Nurses, Public Health”. The searches were performed by one of the researchers (CARS) and verified by a second one (MRÁ) using PRISMA-S for searching extension [15]. The final search strategy established was adapted to each of the databases selected (Supplementary Table S1).

Eligibility criteria: Inclusion criteria, according to the characteristics of the participants: Adult people undergoing grief situations and receiving professional assistance by nurses in primary, home and community care contexts. According to the study designs: Observational studies, both descriptive and analytical (cohort and case–control) and intervention designs, both true experimental (such as RCTs, clinical trials without randomization and without a control group) and quasi-experimental (such as before-after studies). Qualitative studies with any methodology (such as Grounded Theory, Phenomenology and Ethnography) were also included. All studies included were limited to English, Spanish or Portuguese languages. According to the outcomes of the review: Interventions were included which are performed or can be performed by nurses in the PHC setting by means of an individual approach to enhance the grieving process. Exclusion criteria, according to the characteristics of the participants: Studies conducted in the in-hospital clinical settings. According to the study design: Publications that did not correspond to research designs and the gray literature were excluded.

Screening process: Records were exported to an Excel^®^ file for the selection process. After removing duplicates, they were assessed based on titles and abstracts. The full-text documents of the selected records were then retrieved to assess their eligibility according to inclusion and exclusion criteria. The process was carried out independently by two reviewers; a third one was called upon to settle any and all discrepancies. To assess the quality of the studies, the JBI critical appraisal tools appropriate to each research design were used, establishing as a criterion of good quality a score of more than 70% with respect to the items included in each tool. A pilot phase was carried out with a sample of records to verify suitability of the process.

Definition of the study variables: The main research results consisted of identifying the types of interventions which are performed or can be performed by nurses in the PHC setting by means of an individual approach to enhance the grieving process. The secondary results included measurements regarding grief adaptation in caregivers, family members and community nurses.

Data extraction: The bibliometric and sociodemographic variables from the studies included were extracted, as well as the descriptive and statistical information corresponding to the clinical variables related to the interventions to reduce maladaptive grief or maladaptive grief risk in the adult people. Data extraction was carried out independently by two researchers and a third reviewer settled any and all discrepancies. The Mendeley^®^ reference manager was used for data extraction. A pilot phase of the extraction process was performed with a sample of studies.

## 3. Results

**Figure 1:**
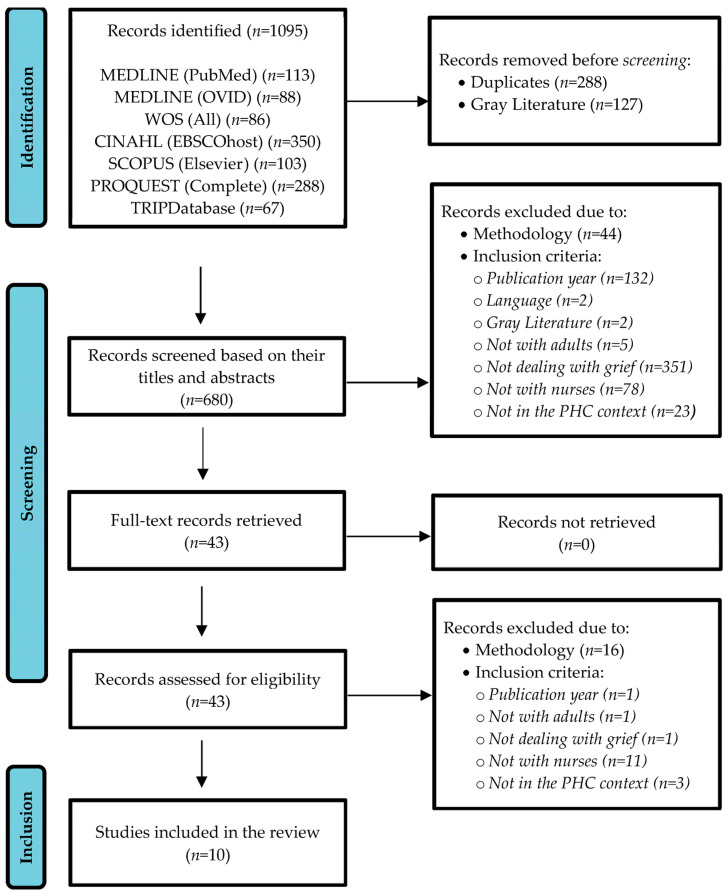
Flowchart of the studies included in the review.

The number of records retrieved was *n* = 1095; after removing duplicates (*n* = 288) and gray literature (*n* = 127), a total of *n* = 680 records were screened based on their titles and abstracts. Of them, *n* = 637 were excluded for not meeting the eligibility criteria, whereas *n* = 43 records did meet the criteria for full-text evaluation. After the critical appraisal process, *n* = 10 studies were included in the review, as shown in the flowchart presented in Figure 1.

Among the studies excluded, *n* = 16 lacked the minimum methodological quality and *n* = 17 did not meet the inclusion criteria (Supplementary Table S2).

After the screening process, the studies included were subjected to a critical appraisal process by two researchers (CARS, MRÁ) who worked independently. For the studies in which 70% agreement was not reached (ref.16,18,20), a third reviewer (PRBB) decided (Supplementary Table S3).

In relation to methodological designs, qualitative (*n* = 4), RCT (*n* = 2), quasi-experimental (*n* = 1), cross-sectional (*n* = 2) and mixed-methods (*n* = 1) studies were included. Table 1 shows the general aspects, objectives, conclusions corresponding to each study, and JBI critical appraisal scores.

In order to organize clinical data extraction, the studies were structured according to their research designs, as follows: qualitative, observational and experimental. The mixed-methods design corresponded both to qualitative and to observational studies for data extraction, following each design.

### 3.1. Qualitative Studies

Among the studies in which professional participants were included, *n* = 3 were community nurses and *n* = 1 were grief counselors; only *n* = 1 study included family members as participants. The topics and subtopics identified are shown in Table 2.

### 3.2. Observational Studies

The observational studies applied different types of instruments. Thus, the mixed-methods study by Hanauer et al. [16] applied an ad hoc questionnaire to observe and descriptively analyze the risks associated with grief during the COVID-19 pandemic. In turn, Song et al. [17] and Ono [24] applied different validated instruments. Song et al. [17] applied the Social Support Rate Scale (SSRS) to assess the differences in relation to social support for grief in urban and rural contexts, as well as PG-13 to know the severity of the grief symptoms in both social environments. Finally, Ono [24] applied several questionnaires to identify and analyze the relationships in grief assistance by community nurses regarding the grief care provided by nurses, observing the outcomes both in the caregivers and in the nurses themselves, as shown in Table 3.

### 3.3. Experimental Studies

The interventions consisted of cognitive–behavioral therapies whose main outcome variable was prolonged grief severity; these studies were developed in different cultural contexts using Psychotherapy overseen by specialists in Psychology. In relation to the RCTs [18,20], they applied the Inventory of Complicated Grief (ICG) instrument, showing statistically significant differences for the interventions. Treml et al. [20] also used the Grief Experience Questionnaire (GEQ) instrument, obtaining significant improvements for the Abandonment/Rejection, Stigmatization, Search for explanations and Guilt dimensions after the intervention. On the other hand, the quasi-experimental study developed by Lichtenthal et al. [21] applied the Prolonged Grief-13 (PG-13) instrument, obtaining a reduction in severity of the grief-related symptoms after the intervention, as shown in Table 4.

## 4. Discussions

This review included studies published after 2009 because this was the year when Nagraj and Barclay [9] conducted their review. Their study was focused on the beliefs of physicians and community nurses about the way in which they cared for their patients during the grieving process. These authors pointed out that neither the physicians nor the nurses wanted to “medicalize” the grieving processes; thus, these care measures represented an important and satisfactory part of their job for which they had received limited training. The studies evaluated by Nagraj and Barclay [9] pointed out that the interventions performed in PHC during grief processes included home visits, phone consultations and condolence letters, concluding that the number of available studies was scarce, that they dated from a minimum of 10 years ago and that they addressed concrete experiences or included small and low-quality samples. In addition, the physicians and the community nurses stated preferences to treat people proactively, although there was in fact a need to improve the assistance provided by developing specific interventions for their clinical practice. The outdated nature of this antecedent justifies our review, for which a scoping design was adopted with the purpose of synthesizing diverse new evidence about the interventions that nurses carry out or can implement in the PHC context by means of an individual approach to reduce maladaptive grief or maladaptive grief risk.

In relation to the qualitative studies, the articles by Robinson et al. [19], Redshaw et al. [2] and Hanauer et al. [16] did not identify the methodological approach in their designs, whereas it was Phenomenology in Johnson [22] and Grounded Theory in Brownhill et al. [23]. These qualitative findings pointed out topics related to social/functional aspects and psych-emotional elements of the families and caregivers, to nurses’ competences and to characteristics of the inter-professional relationships and the health system. Robinson et al. [19] examine the families’ perceptions regarding the end-of-life care provided by nurses. Although they are not specifically focused on grief, they provide a perspective about how the care provided by nurses is perceived and valued, as well as on the need to integrate care in end-of-life assistance, highlighting two important aspects in the care provided by nurses: on the one hand, physical care and care-related administrative tasks and, on the other hand, emotional care and assistance focused on empathy, cultural sensitivity and effective communication. Redshaw et al. [2] and Johnson [22] provide insights about community nurses’ challenges and strengths in terms of grief care continuity. Lack of time, resources and specific training stand out among the challenges. The strengths include nurses’ ability to get involved (both emotionally and with cultural sensitivity) in individualized relationships, highlighting personal connection, mutual understanding, closeness and commitment to the patients, caregivers and families. These circumstances can hinder the debonding process when the relationship is long-term. On the contrary, it is easier to keep an empathic attitude when the bonds with patients and families have been shorter in time, although this can negatively affect the level of grief care [22].

Hanauer et al. [16] provide a specific view of the risk factors for prolonged grief during the COVID-19 pandemic, highlighting the isolation and loneliness experienced by grieving people due to the restrictions and social distancing measures. Through a mixed-methods design, the study also describes the risk factors for prolonged grief, which include losing a loved one due to COVID-19, lack of social support, the presence of other stressful events and the absence of resources to adapt to grief. These findings highlight the importance of an early intervention to mitigate the risk of complications during the grieving process.

Brownhill et al. [23] highlight the importance of addressing grief-related needs in the community context, where nurses can play a crucial role in supporting people with a holistic approach. The main strength of the study by Brownhill et al. [23] consists of an approach that provides structure to a model for decision-making by community nurses grounded on the relationships established, the workloads, the psychosocial aspects, social–family support, the grief circumstances and the mix of different nurse profiles; however, the limitations of the model proposed must be considered due to the fact that grief experiences can vary according to personal, social and cultural factors.

Among the observational studies, only Song et al. [17] studied grieving people by applying the SSRS and PG-13 instruments to assess social support and the severity of the grief-related symptoms, although it should be considered that the answers and the grief sequence/duration are dynamic and individual and can vary from one person to another [25]. The results indicated the importance of grieving people living in urban/rural areas for social support and PGD symptoms in parents that have lost a child. In addition, other factors have been described; Zhou et al. [26] highlighted that younger parental age, being a mother, living in a rural location, lower monthly capital income, shorter time since loss and more comorbid chronic physical conditions were associated with more severe PGD symptoms. Regarding the differences between rural and urban areas, in contrast to our results, Dorsey et al. [27] studied the use of cognitive behavioral therapy (CBT) versus usual care for children in the context of Africa (Kenya and Tanzania), showing that in all groups (rural and urban) CBT was more effective (rural Kenya Cohen d = 1.04 [95% CI, 0.72–1.36]), urban Kenya Cohen d = 0.56 [95% CI, 0.29–0.83]), and urban Tanzania Cohen d = 0.45 [95% CI, 0.10–0.80]). At 12-month follow-up, TCC remained more effective than usual care in both the rural (Cohen d = 0.86 [95% CI, 0.64–1.07]) and urban groups (Cohen d = 0.99 [95% CI, 0.75–1.23]).

On the other hand, according to Xiong et al. [28], parents with social support are better able to manage symptoms of anxiety and stress, thereby reducing the risk of PGD compared to those with less social support. Thus, in the meta-analysis by Yuan et al. [29], the prevalence of PGD was 20.9% (95% CI, 13.8–30.3%), while PGD symptoms were 75.0% (95% CI, 14.9–98.1%). Subgroup analyses highlighted the proportion of mothers in the study samples as a statistically significant heterogeneity factor. Specifically, a significantly higher prevalence of PGD was observed in studies with a percentage of mothers > 60% than in those with a percentage of mothers ≤ 60% (30.9% vs. 14.0%, *p*= 0.001). This result highlights the importance of clinical follow-up of these mothers because of their increased vulnerability.

The other two observational studies included populations comprising professionals. For Hanauer et al. [16], the COVID-19 pandemic has affected grief experiences, the approach to grieving processes and the specific risk factors that allow the provision of the best assistance possible. Moreover, Ono [24] pointed out that nurses should improve their professional competences and skills through training instead of expecting to acquire them through experience. In addition, caring during grieving processes offers nurses the opportunity to receive feedback about the care provided, which motivates them and allows preserving a certain balance in terms of their mental health.

No interventions performed by nurses were included in the experimental studies, showing the existing knowledge gaps in this line of research for Nursing. The available studies consisted of cognitive–behavioral therapies using Psychotherapy overseen by specialists in Psychology and assessed with the ICG, GEQ and PG-13 instruments [18,20,21]. The interventions consisted of several sessions with scheduled tasks to reprocess the traumatic memories and reduce avoidance behaviors, aimed at self-coping and cognitive restructuring, to end up with a presentation to share the lessons learned during the therapeutic process by writing a letter where the grieving person describes the experiences they underwent, as well as the new coping strategies and perspectives.

Although the RCTs conducted by Kaiser et al. [18] and by Treml et al. [20] provide valuable information about these interventions for grief, they present methodological limitations that should be considered to interpret their results. These limitations are due to the small sample size, which cannot be generalized to other population segments, and to an insufficient follow-up time to thoroughly assess long-term effectiveness after the intervention. In relation to the study by Lichtenthal [21], the results point to a reduction in the severity of grief-related symptoms both during and after the intervention. However, the authors present the scores obtained and the effect size without reporting the statistical significance values.

Regarding the use of the Nursing Interventions Classification (NIC) for PGD in the scientific literature, in a previous scoping review conducted by Rodríguez-Suárez et al. [30], no interventions for addressing grief were identified. The potential interventions that might be implemented by nurses and described in the NIC include behavioral therapy ones, which are defined as interventions to strengthen or foster desirable behaviors or modify undesirable ones [26]. Among others, these interventions include reaching a consensus with the patient, assisting in self-change, assertiveness training, training to control impulses, definition of shared objectives, behavioral management, behavioral change, therapy with the environment, activity therapy, game therapy and dance therapy. These NICs might be used by PHC nurses to assess maladaptive grief processes or maladaptive grief risk [31].

Although the authors do not recommend implementing formal or structured interventions due to closeness and to the therapeutic relationships established in people’s family and community contexts, the PHC clinical context seems to be the ideal care level to address grief. Nevertheless, it is currently not possible to issue rigorous evidence-based recommendations regarding care measures for grieving people, except for the pharmacological treatment of depression [5]. On the other hand, grieving people usually do not attend or request appointments with PHC professionals, whose role is to provide information about grief and the types of resources available [5,31].

Age, type of relationship with the deceased person, death circumstances, lack of social support and previous unresolved grief are among the known risk factors for maladaptive grief. On the other hand, the most researched protective factors are resilience, spirituality and safe attachment [32]. Consequently, Rodríguez-Álvaro et al. [33] pointed out that the predominating family caregiver profile in the Canary Islands corresponds to 60-year-old women with anxiety or depression problems. According to these authors, the most extended traditional grief theories are based on female coping styles, a possible reason for why it might be easier for professionals to identify clinical manifestations of grief in women than in men. They also note that women talk, reflect and participate more actively in interviews and meetings than men while elaborating their grief. In addition, according to these authors, it should be considered that if the deceased person is a spouse, the life event is perceived as extremely stressful and the impact on health is very high.

The limitations of this research are related to the absence of studies that describe or analyze specific interventions with nurses in the PHC context. The limitations due to the methodological time filter should be noted, as only studies published after 2009 were included. Additionally, there is a linguistic bias since only studies published in English, Spanish, and Portuguese were considered. This bias could be significant, given that many studies have been published in the context of Asia.

Furthermore, the existing studies with experimental designs are restricted to a few Psychotherapy interventions developed by psychologists, whereas the qualitative and observational studies available have been mostly developed for populations comprising professionals, focusing less on the results about grief severity in grieving people and families. In the future, it will be essential to conduct a more in-depth investigation into the differences in PGD between rural and urban settings across a wider range of international contexts. Additionally, further research is needed to explore how different subgroups, such as gender or age, respond to interventions designed to improve grief.

## 5. Conclusions

The diverse evidence retrieved from the studies that address the reduction in maladaptive grief or maladaptive grief risk by means of individual Nursing interventions in the PHC context is not conclusive. There is a need to increase both the number and the methodological quality of studies assessing the effectiveness of Nursing care in PHC for individuals experiencing maladaptive grief or maladaptive grief risk. Further research should focus on experimental studies, developing specific interventions conducted by nurses to address individual’s grief and prevent maladaptive grief. As implications for clinical practice, this review allows identifying knowledge gaps in the grief assistance scope in the PHC setting, with the need to develop more rigorous studies with specific interventions implemented by community nurses to address grief in an individualized way and prevent maladaptive grief.

## Figures and Tables

**Table 1:** General aspects of the studies included.

Author (Year) Country Design	Objectives	Conclusions	JBI Score (%)
Hanauer et al. (2023) [16] Germany Mixed methods (Qualitative: no specified approach. Quantitative: cross-sectional)	To identify and analyze the relationships between the grief assistance provided by nurses and the grief care results.	The pandemic has affected grief experiences and counseling on this subject matter. Counselors should monitor the grieving process and the specific risk factors to provide the best assistance possible to grieving people when necessary.	88.9/50
Song et al. (2023) [17] China Cross-sectional	To assess the prevalence of complicated grief symptoms among Chinese shiduers * and to investigate the association between social support and complicated grief symptoms in urban and rural areas.	The results revealed high rates of PGD ^a^ symptoms in Chinese shiduers; the findings highlight the important role of urban/rural location in the relationship between social support and PGD symptoms.	100
Kaiser et al. (2022) [18] Germany RCT	To deepen on the previously obtained findings by assessing a web-based cognitive–behavioral intervention with asynchronous support by the therapist, consisting of structured writing tasks specifically adapted to prolonged grief due to cancer.	The web-based intervention for prolonged grief due to cancer is effective to reduce PGD symptoms and those of the accompanying syndromes in a timely and easy-to-implement way, in addition to addressing the specific challenges inherent to grief due to a disease. Considering web-based approaches in future mental health care policies and practices may reduce the gaps in the health assistance provided to grieving people due to cancer.	69.2
Robinson et al. (2022) [19] New Zealand Qualitative (no specified approach)	To explore the grieving families’ perceptions and experiences about community nurses in end-of-life care, with a special focus on service integration.	The results support the need for a new Nursing model of integrated Palliative Care that resorts to the set of unique skills of nurses working in community care, including general Nursing, Palliative Care and community care services. By adapting different Nursing care models, it is possible to meet the patients’ needs instead of merely limiting the actions to a defined model for service provision.	77.8
Treml et al. (2021) [20] Germany RCT	To examine the efficacy of cognitive–behavioral therapy for grief based on Internet use specifically for grieving people due to suicide.	The cognitive–behavioral therapy for grief based on Internet use represents an effective treatment modality for people undergoing prolonged grief after a loss due to suicide. Considering the effect sizes, the brief treatments and stability of the results, it constitutes an appropriate alternative for in-person grief interventions.	69.2
Lichtenthal et al. (2019) [21] Australia Quasi-experimental	To determine preliminary feasibility, acceptability and the effects of grief therapy focused on meaning for parents that have lost a child due to cancer.	In general, the preliminary data suggest that this cognitive–behavioral–existential intervention implemented in 16 sessions is feasible and acceptable and is associated with cross-diagnostic improvements in the psychological functioning of parents that have lost a child due to cancer. Future research studies should examine grief therapy focused on meaning with a larger sample in an RCT ^b^.	77.8
Johnson (2015) [22] UK Qualitative (Phenomenology)	To explore how grief assistance is provided from the perspective of community nurses.	Grief assistance is part of nurses’ role; the results suggest that some nurses get too involved without being able to set a resolution date, as each case depends on specific needs. Expanded experience in grief assistance and exposure to grief increased awareness regarding its importance and improved confidence in the care provided.	100
Brownhill et al. (2013) [23] Australia Qualitative (Grounded Theory)	To perform an in-depth analysis of an existing dataset generated from semi-structured interviews with 10 community nurses that carried out grief assistance follow-up home visits in an area health service from a metropolitan region of Sydney, Australia.	An in-depth exploration of the decision-making process in grief assistance has given rise to a decision model that explicitly states the community nurses’ assessments of one alternative over the other. The results highlight the complexity of the community nurses’ role, especially when decision-making is discretionary and depends on multiple variables in a context marked by uncertainty. We expect that this model may provide important information to service providers and improve the role of other community nurses in grief assistance, in addition to improving the outcomes for grieving people.	77.8
Ono (2013) [24] Japan Cross-sectional	To identify the relationships inherent to the grief assistance provided by community nurses.	It is not enough to be told how to care for a patient in the terminal phase. The care measures during this period seem to require years of experience as a community nurse. Nurses should consider more effective educational methods to foster this skill instead of expecting to acquire it through experience. Implementing grief care is an opportunity to receive feedback about the assistance provided by nurses. Grief care is a motivation source for nurses and preserves their mental health.	87.5
Redshaw et al. (2013) [2] Australia Qualitative (no specified approach)	To explore community nurses’ perceptions regarding the grief care they provide to their patients, caregivers and family members.	The study contributes specific evidence for adopting a model of the grief support provided by community nurses as a means to reduce the chances of complicated grieving processes in the community. In addition, home visits provide an important opportunity for nurses to satisfactorily finish their relationship.	77.8

* Shiduers: Adults that have lost a child; ^a^ PGD: Prolonged Grief Disorder; ^b^ RCT: Randomized Control Trial.

**Table 2:** Qualitative studies included.

Author (Year) Participants	Topics	Subtopics
Hanauer et al. (2023) [16] 30 grief counselors	- Absence of social support - Routines lost - Additional stress factors - Social impact of the pandemic - Personal values and priorities - Grief support/health care impact	
Robinson et al. (2022) [19] 23 family members	Perception regarding the nurses’ roles and the care provision services	- Physical care measures: community nurses’ experiences - Talking about care: Palliative Care hospital nurses’ experiences - I did not expect that she would come to my house - Experiences with general care nurses
	Experiences with the integration of Nursing services	
Johnson (2015) [22] 5 community nurses	Time management	- Time consumption/Prolonged periods - Capacity/Workload/Balance - Follow-up call (phone/visit)
	Specific/Collateral cases	- Relationships/Involvement - Attitudes/Approach - Individualized assessments - Younger families
	Inter-professional work	- Greater sensitization - Guidelines (Health Department, Health and Care Excellence National Institute) - Communication
	Role/Scope	- Skill/Capability - Challenging/Exhausting/Comprehensive
	Experience/Learning	- Training/Theory (ongoing)/Skills - Confidence/Exposure/Reinforcement - New knowledge - Empathy/Interest
Brownhill et al. (2013) [23] 10 community nurses	It depends on…	- The relationships - The workloads - The psychosocial aspects - The support - The circumstances - The mix of nurses
Redshaw et al. (2013) [2] 10 community nurses	Allow caregivers to be the core of Nursing care	
	Provide caregivers with a way out after death	
	Provide nurses with the opportunity to finish a relationship with the caregiver and the deceased	

**Table 3:** Observational studies included.

Author; Year	Population and Sample	Instruments	Descriptive Results	Inferential Results
Hanauer et al., 2023 [16]	*n* = 93 48.4% full-time counselors	Online ad hoc survey. Developed from a non-systematic search for 8 risk factors associated with grief during the COVID-19 pandemic.	- Limited possibilities to visit/be with a dying person (94.4%) - Absence of social support (94.4%). - Absence of grief-related traditional rituals (92.1%) - Useful routines/distractions lost or more focus on grief (78.4%). - Traumatic circumstances inherent to death (68.5%) - Additional stress factors (67.8%). - Truncated assumptions and beliefs about oneself, the environment and the future (52.8%). - Increased death anxiety or coping with own mortality (37.5%).	
Song et al., 2023 [17]	*n* = 405	SSRS ^a^: 10 items 3 dimensions for social support: Subjective support (α = 0.706) Objective support (α = 0.713) Support availability (α = 0.671) PG-13 ^b^: 13 items; α = 0.861 4 dimensions: - Separation distress. - Duration criteria. - Cognitive, emotional and behavioral symptoms. - Functional impairment criteria. 5-point Likert scale PGD criteria: At least 1 item with a score equal to or greater than 4 in the “Separation distress” dimension. At least 5 items with a score equal to or greater than 4 in the “Cognitive, emotional and behavioral symptoms” dimension. 2 items with affirmative answers in the “Duration criteria” and “Functional impairment criteria” dimensions. The higher the value added up in the first 11 items, the more severe the symptoms.	SSRS: Social support (Mean = 31.08; SD = 6.25) Subjective support (Mean = 17.51; SD = 3.52) Objective support (Mean = 7.17; SD = 2.68) Support availability (Mean = 6.39; SD = 1.65) PG-13: Mean = 36.19 (SD = 9.19) Range (12–55) PGD ^c^ criteria N = 120 (29.63%) PGD symptoms (Mean = 36.19; SD = 9.18) PGD Urban Shiduers N = 237 (Mean = 34.93; SD = 9.25) PGD Rural Shiuders N = 167 (Mean = 37.94; SD = 8.80) - *t* test (*p* = 3.291).	The “urban/rural location” variable was positively associated with PGD symptoms (β = 0.253; *p* < 0.01). Multiple regression analysis (impact of urban/rural location and social support on PGD symptoms): Objective support (β = 0.183; *p* < 0.01), subjective support (β = 0.207; *p* < 0.01) and support availability (β = 0.202; *p* < 0.01) were negatively related to PGD symptoms. Significant interaction effect for objective support and urban/rural location (β = 0.176; *p* < 0.01) but not for subjective support and urban/rural location (β = 0.051; *p* < 0.05) or for support availability and urban/rural location (β = 0.081; *p* < 0.05). (Urban/rural location moderates the effect of objective support on the shiduers’ PGD symptoms; in turn, there is no significant effect in the relationship of subjective support and support availability in terms of PGD symptoms.) Objective support exerted a significantly negative predictive effect on PGD symptoms among rural *shiduers* (β = 1.182; *t* = 4.592; *p* < 0.01) but showed no significant effect among their urban counterparts (β = 0.249; *t* = 1.357; *p* < 0.05).
Ono, 2013 [24]	*n* = 332 managers *n* = 1442 staff members	Grief Care Provided by Nurses Scale. Likert from 1 (Never provided it) to 5 (Always provided it) 3 subscales: (1) GCBT ^d^ 16 items; α = 0.933 dimensions (α = 0.87–0.91): - Promotion of death acceptance and explanation of the death assistance system. - Support for family care continuation with respect to their intent. - Empathy for the family’s feelings. Mean scale 62.9 (SD = 11.0; Range = 16–80) (2) GCDB ^e^ 5 items; α = 0.66 Mean scale 21.0 (SD = 3.3; Range = 7–25) (3) GCAD ^f^ 21 items; α = 0.81 3 dimensions (α = 0.85–0.89) - Sharing and support of the family’s experience regarding the parent’s death. - Psychological support for rebuilding life - Grasping of state for resuming social activities. Mean scale 71.4 (SD = 15.2; Range = 23–105) Family Caregivers’ Outcomes Scale 19 items; α = 0.81 4 dimensions (α = 0.72–0.82): - Acquisition of positive feelings and grief alleviation. - Expansion of social roles. - Negative effect of grief care. - Prevention of illness and death due to influence of bereavement. Likert from 1 (It does not apply) to 5 (It applies very much). Mean scale 65.9 (SD = 7.5; Range = 40–95) Nurses’ Outcomes Scale 13 items; α = 0.73 4 dimensions (α = 0.76–0.80): - Learning opportunity. - Negative psychological effect. - Confidence in visiting Nursing. - Deepening of trusting relations at the workplace. Likert from 1 (It does not apply) to 5 (It applies very much). Mean scale 46.8 (SD = 5.4; Range = 29–65)	Grief Care Provided by Nurses Scale. GCBT (*n* = 675) GCDB (*n* = 399) GCAD (*n* = 543) The descriptive results were not reported Family Caregivers’ Outcomes Scale - Acquisition of positive feelings and grief alleviation (Mean = 22.3; SD = 3.3 Range = 6–30). - Expansion of social roles (Mean = 16.1; SD = 2.7; Range = 5–25). - Prevention of illness and death due to influence of bereavement (Mean = 10.4; SD = 2.1; Range = 3–15). Nurses’ Outcomes Scale - Learning opportunity (Mean = 16.3; SD = 2.5; Range = 6–20). - Negative psychological effect (Mean = 13.6; SD = 3.5; Range = 4–20). Deepening of trusting relations at the workplace (Mean = 8.0; SD = 1.5; Range = 2–10)	Grief Care Provided by Nurses Scale. GCBT: Nurses’ significant personal factors: - Years of experience as a home-care nurse (β = 0.102; *p* < 0.01). - Number of treatments after the death/year (β = 0.200; *p* < 0.001). - Number of home visits after the patient’s death/year (β = 0.105; *p* < 0.05). - Experience in learning grief assistance after the patient’s death (β = 0.110; *p* < 0.01). Nurses’ significant environmental factors: - The duty to provide grief assistance after a death as part of their job (β = 0.074; *p* < 0.05) GCDB: Nurses’ significant personal factors: - Number of treatments after the death/year (β = 0.200; *p* < 0.001). - Experience in learning grief assistance after the patient’s death (β = 0.111; *p* < 0.05). Nurses’ significant environmental factors: - The duty to provide grief assistance after a death as part of their job (β = 0.138; *p* < 0.01). GCAD: Nurses’ significant personal factors: - Experience in learning grief assistance after the patient’s death (β = 0.135; *p* < 0.01). Family Caregivers’ Outcomes Scale Acquisition of positive feelings and grief alleviation: - GCBT (β = 0.241; *p* < 0.001). - GCAD (β = 0.109; *p* < 0.05). Expansion of social roles: - GCAD (β = 0.299; *p* < 0.001). Prevention of illness and death due to influence of bereavement: - GCBT (β = 0.143; *p* < 0.05). - GCAD (β = 0.124; *p* < 0.05). Nurses’ Outcomes Scale Learning opportunity: - GCBT (β = 0.177; *p* < 0.01). - GCDB (β = 0.180; *p* < 0.01). - GCAD (β = 0.151; *p* < 0.01). Negative psychological effect: - GCAD (β = 0.183; *p* < 0.01). Deepening of trusting relations at the workplace: - GCBT (β = 0.133; *p* < 0.05). GCDB (β = 0.178; *p* < 0.01)

^a^ SSRS: Social Support Rate Scale; ^b^ PG-13: Prolonged Grief-13; ^c^ PGD: Prolonged Grief Disorder; ^d^ GCBT: Grief Care from Beginning of Home Care to the Terminal Period; ^e^ GCDB: Grief Care at Deathbed; ^f^ GCAD: Grief Care After the Patient’s Death.

**Table 4:** Experimental studies included (outcome = prolonged grief severity).

Author; Year	Population and Sample	Intervention	Instruments	Mean (SD) Baseline	Before and After the Intervention Mean (SD)	Effect Size
Kaiser et al., 2022 [18]	*n* = 87 IG ^a^: 44 (5 losses) CG ^b^: 43 (1 loss)	Online Grief Therapy. (Three modules: Self-coping, Cognitive restructuring, Social exchange). It was applied in two sessions (one each week) with 10 written tasks (45 min)	ICG ^c^ (German version) 19 items; α = 0.82	Total: 37.94 (10.27) IG: 38.98 (9.87) CG: 36.88 (10.67) *p* = 0.35	Before: IG: 39.0 (9.9) CG: 36.9 (10.7) After: IG: 27.5 (10.4) CG: 36.0 (10.8)	Intragroup: IG: f: 58.9; *p* < 0.001 CG: f: 0.9; *p* = 0.34 Intergroup: f: 40.7; *p* < 0.001 η2: 0.35 (0.20–0.46) d: 0.80 (0.35–1.25)
Treml et al., 2021 [20]	*n* = 58 IG: 30 CG: 28	Online Cognitive Behavioral Grief Therapy. Three phases with 10 written tasks: Self-coping (describing emotional and sensory thoughts and perceptions), Cognitive restructuring (writing a support letter to a friend that has suffered the same loss, including feelings of guilt, anger or shame) and Social exchange (writing a final letter to summarize and share the lessons learned during the therapeutic process). Each phase included psychoeducation on the meaning and antecedents of the treatment technique.	ICG: 19 items; α = 0.83 GEQ ^d^: 55 items;α = 0.92 (Total GEQ) 8 dimensions: - Somatic reactions - Abandonment/Rejection - Stigmatization - Search for explanations - Guilt - Responsibility - Shame/Embarrassment (α = 0.73–0.88) - Self-destructive behavior (α = 0.59) 5-point Likert scale(Range: 55–275)	ICG: Total sample: 35.90 (10.34) IG: 35.43 (10.57) CG: 36.40 (10.25) *p* = 0.73 Total GEQ: Total sample: 151.05 (32.39) IG: 151.97 (33.40) CG: 150.07 (31.85) *p* = 0.83	ICG Before: IG: 35.43 (11.19) CG: 36.39 (11.19) After: IG: 24.79 (11.47) CG: 36.72 (11.29) Total GEQ Before: IG: 151.97 (34.10) CG: 150.07 (34.10) After: IG: 129.77 (34.80) CG: 150.15 (34.34) Somatic reactions Before: IG: 7.80 (3.46) CG: 8.82 (3.46) After: IG: 6.87 (3.54) CG: 8.69 (3.50) Abandonment/Rejection Before: IG: 32.73 (8.42) CG: 31.39 (8.42) After: IG: 27.54 (8.56) CG: 32.34 (8.49) Stigmatization Before: IG: 30.97 (9.56) CG: 30.36 (9.56) After: IG: 26.68 (9.68) CG: 29.53 (9.60) Search for explanations Before: IG: 25.00 (6.52) CG: 25.82 (6.52) After: IG: 19.36 (6.63) CG: 25.92 (6.58) Guilt Before: IG: 20.30 (6.74) CG: 17.82 (6.74) After: IG: 16.22 (6.83) CG: 17.58 (6.79) Responsibility Before: IG: 11.07 (5.36) CG: 11.64 (5.36) After: IG: 10.03 (5.45) CG: 11.25 (5.41) Shame/Embarrassment Before: IG: 7.70 (2.78) CG: 8.03 (2.78) After: IG: 6.90 (2.83) CG: 7.95 (2.80) Self-destructive behavior Before: IG: 7.70 (2.78) CG: 8.03 (2.78) After: IG: 6.90 (2.83) CG: 7.95 (2.80)	ICG: d: 0.97; *p* < 0.001 Total GEQ: d: 0.65; *p* = 0.002 Somatic reactions: d: 0.23; *p* = 0.335 Abandonment/Rejection: d: 0.61; *p* = 0.002 Stigmatization: d: 0.36; *p* = 0.019 Search for explanations: d: 0.87; *p* < 0.001 Guilt: d: 0.56; *p* = 0.005 Responsibility d: 0.12; *p* = 0.739 Shame/Embarrassment d: 0.09; *p* = 0.681 Self-destructive behavior d: 0.26; *p* = 0.125
Lichtenthal et al., 2019 [21]	*n* = 8	Grief therapy focused on meaning (parents that have lost a child due to cancer) 16 sessions (60–90 min) (video conference) Individualized and manualized cognitive–behavioral–experiential–existential intervention that resorts to psychoeducation, structured debate and experiential exercises focused on topics related to meaning, identity, purpose and legacy.	PG-13 ^e^ It assesses the frequency of 4 grief-related symptoms in the last month and the severity of 7 current grief-related symptoms. Other items assess the duration of the symptoms and functional impairment. 5-point Likert-type scale. Scores from 11 to 55 (high scores indicate severe PGD ^f^ symptoms).	39.50 (6.1) Range (30–49)	M2 (intervention midpoint): Mean difference: −3.14 (3.0) M3 (post-intervention) Mean difference: −8.17 (4.8) M4 (3 months after the intervention): Mean difference: −7.33 (5.8)	M2: d = −1.06 M3: d = −1.70 M4: d = −1.26 *p*-value not reported

^a^ IG: Intervention Group; ^b^ CG: Control Group; ^c^ ICG: Inventory of Complicated Grief; ^d^ GEQ: Grief Experience Questionnaire; ^e^ PG-13: Prolonged Grief-13; ^f^ PGD: Prolonged Grief Disorder.

## Supplementary Materials

The following supporting information can be downloaded at: https://www.mdpi.com/article/10.3390/nursrep14030178/s1, Table S1: Search strategies, Table S2: Excluded studies from the review, Table S3: Critical appraisal process of the included studies.

## Public Involvement Statement

There was no public involvement in any aspect of this research.

## Guidelines and Standards Statement

This researched used the criteria set forth in the Preferred Reporting Items for Systematic Reviews and Meta-Analyses extension for Scoping Reviews (PRISMA-ScR) and in Enhancing Transparency in Reporting the Synthesis of Qualitative Research (ENTREQ). The review protocol has been registered and is available in https://osf.io/gawjr/ (accessed on 12 February 2023) (DOI: 10.17605/OSF.IO/GAWJR).
