# Supplementary material for: Nursing Interventions in Primary Care for the Management of Maladaptive Grief: A Scoping Review
Source: Nurs Rep. 2024 Sep 14;14(3):2398–414. doi: 10.3390/nursrep14030178 (PMC11417849; doi:10.3390/nursrep14030178)
Supplement: Supplementary file 1 [file nursrep-14-00178-s001.zip › Supplementary Table S1.pdf]

Supplementary Table S1. Search strategies.

Database<br>Date	Search strategies
Medline (PubMed)<br>11/25/2023	Search: (((("Disenfranchised Grief"[Mesh] OR "Grief, Disenfranchised" OR "Hidden Grief") OR ("Grief"[Mesh] OR "Griefs" OR "Mourning" OR "Mournings")) OR ("Bereavement"[Mesh] OR "Bereavements")) OR ("Prolonged Grief Disorder"[Mesh] OR "Disorder, Prolonged Grief" OR "Grief Disorder, Prolonged" OR "Prolonged Grief Disorders")) AND ((((("Primary Care Nursing"[Mesh] OR "Care Nursing, Primary" OR "Nursing, Primary Care") OR ("Community Health Nursing"[Mesh] OR "Nursing, Community Health")) OR ("Home Health Nursing"[Mesh] OR "Nursing, Home Health" OR "Home Health Care Nursing")) OR ("Nurses, Community Health"[Mesh] OR "Community Health Nurse" OR "Community Health Nurses" OR "Nurse, Community Health" OR "Visiting Nurses" OR "Nurse, Visiting" OR "Nurses, Visiting" OR "Visiting Nurse" OR "Home Health Nurses" OR "Home Health Nurse" OR "Nurse, Home Health" OR "Nurses, Home Health" OR "Home Nurses" OR "Home Nurse" OR "Nurse, Home" OR "Nurses, Home" OR "Health Visitors" OR "Health Visitor")) OR ("Nurses, Public Health"[Mesh] OR "Nurse, Public Health" OR "Public Health Nurse" OR "Public Health Nurses"))
Medline (OVID)<br>11/25/2023	(("Primary Care Nursing" or "Care Nursing, Primary" or "Nursing, Primary Care" or "Community Health Nursing" or "Nursing, Community Health") and ("Prolonged Grief Disorder" or "Disorder, Prolonged Grief" or "Grief Disorder, Prolonged" or "Prolonged Grief Disorders" or "Bereavement" or "Bereavements" or "Grief" or "Griefs" or "Mourning" or "Mournings"))
ProQuest (Complete)<br>11/25/2023	title("Primary Care Nursing") OR abstract("Primary Care Nursing") OR title("Community Health Nursing") OR abstract("Community Health Nursing") AND title("Prolonged Grief Disorder") OR abstract("Prolonged Grief Disorder") NOT MEDLINE®/PEER-REVIEWED ARTICLES/FULLTEXT
Web of Science (All)<br>11/30/2023	TS=("Prolonged Grief Disorder" OR "Bereavement" OR "Grief") AND TS=("Primary Care Nursing" OR "Community Health Nursing")
TripDatabase<br>11/30/2023	("prolonged grief disorder" OR "bereavement" OR "grief") AND ("primary care nursing" OR "community health nursing")
Cinahl (EBSCOhost)<br>12/5/2023	(“Primary Care Nursing” OR “Community Health Nursing”) AND (“Prolonged Grief Disorder”)
Scopus (Elsevier)<br>12/5/2023	TITLE-ABS-KEY ("Prolonged Grief Disorder") OR TITLE-ABS-KEY ("Bereavement") OR TITLE-ABS-KEY ("Grief") AND TITLE-ABS-KEY ("Primary Care Nursing") OR TITLE-ABS-KEY ("Community Health Nursing")
