# Supplementary material for: Nursing Interventions in Primary Care for the Management of Maladaptive Grief: A Scoping Review
Source: Nurs Rep. 2024 Sep 14;14(3):2398–414. doi: 10.3390/nursrep14030178 (PMC11417849; doi:10.3390/nursrep14030178)
Supplement: Supplementary file 1 [file nursrep-14-00178-s001.zip › Supplementary Table S2.pdf]

Supplementary Table S2. Excluded studies from the review

ID	Authors	Title	Journal	Year	DOI	Reason for exclusion
**ID4**	Carson	Walking our homebound patients through terror, grief, and loss	Home Healthc Nurse	2001	10.1097/00004045-200112000-00003	Methodology
**ID3**	Costello & Christoforou	Palliative care in a Mediterranean culture: a review of services in the Republic of Cyprus	Int J Palliat Nurs	2001	10.12968/ijpn.2001.7.6.9026	Methodology
**ID15**	Lyttle	Bereavement visiting: older people's and nurses' experiences.	British journal of community nursing	2001	10.12968/bjcn.2001.6.12.9448	Year of publication
**ID5**	Langridge	Reduction of chronic sorrow: a health promotion role for children's community nurses?	J Child Health Care	2002	10.1177/136749350200600302	Methodology
**ID16**	Weinstein	Bereaved Orthodox Jewish families and their community: a cross-cultural perspective	J Community Health Nurs	2003	10.1207/S15327655JCHN2004_04	Methodology
**ID11**	Robinson	A personal exploration of the power of poetry in palliative care, loss and bereavement	International Journal of Palliative Nursing	2004	10.12968/ijpn.2004.10.1.12017	Methodology
**ID17**	Tuffrey, Finlay &, Lewis	The needs of children and their families at end of life: an analysis of community nursing practice	Int J Palliat Nurs	2007	10.12968/ijpn.2007.13.2.23071	No adults
**ID18**	Onrust et al	Cost-utility of a visiting service for older widowed individuals: randomised trial	BMC Health Serv Res	2008	10.1186/1472-6963-8-128	No nurses
**ID19**	Brito-Brito, Rodríguez-Ramo & Pérez-García-Talavera	Clinical case: complicated grief in primary care. Care plan	Enfermería Clínica	2009	10.1016/j.enfcli.2009.07.011	Methodology
**ID20**	Mathiews	Death with dignity	Creat Nurs	2010	10.1891/1078-4535.16.4.185	Methodology
**ID6**	Ishii, Miyashita, Sato & Ozawa	Family's difficulty scale in end-of-life home care: a new measure of the family's difficulties in caring for patients with cancer at the end of life at home from bereaved family's perspective	J Palliat Med	2012	10.1089/jpm.2011.0248	No nurses

**ID7**	Kim, Lee & Shimanouchi	Needs for end-of-life care by home care nurses among non-cancer patients in Korea and Japan	INTERNATIONAL JOURNAL OF NURSING PRACTICE	2014	10.1111/ijn.12156	Methodology
**ID38**	Li, Prigerson & Holly	Assessment and associated features of prolonged grief disorder among Chinese bereaved individuals	Comprehensive Psychiatry	2016	10.1016/j.comppsych.2015.12.001	No primary health care
**ID12**	Stastny, Keens & Alkon	Supporting SIDS Families: The Public Health Nurse SIDS Home Visit	Public Health Nurs	2016	10.1111/phn.12251	Methodology
**ID28**	AAA Manik et al	Early indicators of problematic grief trajectories following bereavement	European Journal of Psychotraumatology	2017	10.1080/20008198.2018.1423825	No nurses
**ID29**	Bryant et al	Predictors of treatment response for cognitive behaviour therapy for prolonged grief disorder	European Journal of Psychotraumatology	2017	10.1080/20008198.2018.1556551	Methodology
**ID13**	Cheung, Lee & Yip	The associations between religion, bereavement and depression among Hong Kong nurses	BMC Res Notes	2017	10.1186/s13104-017-2588-7	Non-griefing in patients
**ID27**	Heeke, Stammel, Heinrich, & Knaevelsrud	Conflict-related trauma and bereavement: exploring differential symptom profiles of prolonged grief and posttraumatic stress disorder	BMC Psychiatry	2017	10.1186/s12888-017-1286-2	No nurses
**ID30**	Killikelly & Maercker	Prolonged grief disorder for ICD-11: the primacy of clinical utility and international applicability	European Journal of Psychotraumatology	2017	10.1080/20008198.2018.1476441	Methodology
**ID25**	Mendes	A community nurse's role in supporting a 'good' experience of grief	Br J Community Nurs	2017	10.12968/bjcn.2017.22.11.566	Methodology
**ID31**	Milman et al	Prolonged grief symptomatology following violent loss: the mediating role of meaning	European Journal of Psychotraumatology	2017	10.1080/20008198.2018.1503522	No nurses
**ID43**	AAA Manik et al	Do prolonged grief disorder symptoms predict post-traumatic stress disorder symptoms following bereavement? A cross-lagged analysis	Comprehensive Psychiatry	2018	10.1016/j.comppsych.2017.09.001	No primary health care
**ID8**	Nyatanga	Loss, grief and bereavement: an inescapable link in palliative care	Br J Community Nurs	2018	10.12968/bjcn.2018.23.2.70	Methodology

**ID33**	Comtesse & Rosner	Prolonged grief disorder among asylum seekers in Germany: the influence of losses and residence status	European Journal of Psychotraumatology	2019	10.1080/20008198.2019.1591330	No nurses
**ID9**	Reblin et al	Communication of emotion in home hospice cancer care: Implications for spouse caregiver depression into bereavement	Psychooncology	2019	10.1002/pon.5064	Methodology
**ID32**	Steil et al	Prevalence of prolonged grief disorder in a sample of female refugees	BMC Psychiatry	2019	10.1186/s12888-019-2136-1	No nurses
**ID1**	Wenn et al	A pilot randomised controlled trial of metacognitive therapy for prolonged grief	BMJ Open	2019	10.1136/bmjopen-2017-021409	No nurses
**ID39**	Zhang et al	Prolonged grief disorder in Chinese Shidu parents who have lost their only child	European Journal of Psychotraumatology	2020	10.1080/20008198.2020.1726071	No nurses
**ID35**	Chen & Tang	Profiles of grief, post-traumatic stress, and post-traumatic growth among people bereaved due to COVID-19	European Journal of Psychotraumatology	2021	10.1080/20008198.2021.1947563	No primary health care
**ID2**	Elinger et al	Narrative reconstruction therapy for prolonged grief disorder – a pilot study	European Journal of Psychotraumatology	2021	10.1080/20008198.2021.1896126	No nurses
**ID44**	Lund	Prolonged Grief Disorder: An Implementation Gone Awry and a Researcher Going Gonzo	International Journal of Qualitative Methods	2021	10.1177/16094069211020176	Methodology
**ID37**	Sardella et al	Prolonged Grief Disorder and Symptoms of Anxiety and Depression among Bereaved Family Caregivers in the Context of Palliative Home Care	European Journal of Investigation in Health, Psychology and Education	2023	10.3390/ejihpe13020037	No nurses
