# Supplementary material for: Nursing Interventions in Primary Care for the Management of Maladaptive Grief: A Scoping Review
Source: Nurs Rep. 2024 Sep 14;14(3):2398–414. doi: 10.3390/nursrep14030178 (PMC11417849; doi:10.3390/nursrep14030178)
Supplement: Supplementary file 1 [file nursrep-14-00178-s001.zip › Supplementary Table S3.pdf]

Supplementary Table S3. Inter-observer agreement for the studies included.

Author; year	Design	Agreement %	Q1*	Q2*	Q3*	Q4*	Q5*	Q6*	Q7*	Q8*	Q9*	Q10*	Q11*	Q12*	Q13*	3rd Reviewer
Hanauer et al.; 2023 [16]	Mixed-methods	88.9/50	Y[a]/N[b]	Y/Y	Y/N	Y/N	Y/Y	Y/Y	Y/Y	Y/N	N/-					Y
Song et al.; 2023 [17]	Cross-sectional	100	Y	Y	Y	Y	Y	Y	Y	Y	Y					
Kaiser et al.; 2022 [18]	RCT	69.2	N	Y	Y	N	N	Y	Y	Y	Y	Y	Y	Y	N	Y
Robinson et al.; 2022 [19]	Qualitative	77.8	Y	Y	Y	Y	N	Y	Y	Y	N					
Treml et al.; 2021 [20]	RCT	69.2	N	Y	Y	Y	N	N	Y	Y	Y	Y	Y	Y	N	Y
Lichtenthal et al.; 2019 [21]	Quasi experimental	77.8	Y	N	N	Y	Y	Y	Y	Y	Y					
Johnson; 2015 [22]	Qualitative	100	Y	Y	Y	Y	Y	Y	Y	Y	Y					
Brownhill et al.; 2013 [23]	Qualitative	77.8	Y	Y	Y	Y	Y	Y	Y	N	N					
Ono; 2013 [24]	Cross-sectional	87.5	Y	Y	N	Y	Y	Y	Y	Y						
Redshaw et al.; 2013 [25]	Qualitative	77.8	Y	Y	Y	Y	Y	Y	N	Y	N					

[a]Y: Yes; [b]N: No

*Quasi-experimental (Q1: Is it clear in the study what is the 'cause' and what is the 'effect' (i.e. there is no confusion about which variable comes first)?; Q2: Were the participants included in any comparisons similar?; Q3: Were the participants included in any comparisons receiving similar treatment/care, other than the exposure or intervention of interest?; Q4: Was there a control group?; Q5: Were there multiple measurements of the outcome both pre and post the intervention/exposure?; Q6: Was follow up complete and if not, were differences between groups in terms of their follow up adequately described and analyzed?; Q7: Were the outcomes of participants included in any comparisons measured in the same way?; Q8: Were outcomes measured in a reliable way?; Q9: Was appropriate statistical analysis used?).

*Qualitative (Q1: Is there congruity between the stated philosophical perspective and the research methodology?; Q2: Is there congruity between the research methodology and the research question or objectives?; Q3: Is there congruity between the research methodology and the methods used to collect data?; Q4: Is there congruity between the research methodology and the representation and analysis of data?; Q5: Is there congruity between the research methodology and the interpretation of results?; Q6: Is there a statement locating the researcher culturally or theoretically?; Q7: Is the influence of the researcher on the research, and vice- versa, addressed?; Q8: Are participants, and their voices, adequately represented?; Q9: Is the research ethical according to current criteria or, for recent studies, and is there evidence of ethical approval by an appropriate body?; Q10: Do the conclusions drawn in the research report flow from the analysis, or interpretation, of the data?).

*Cross-sectional (Q1: Were the criteria for inclusion in the sample clearly defined?; Q2: Were the study subjects and the setting described in detail?; Q3: Was the exposure measured in a valid and reliable way?; Q4: Were objective, standard criteria used for measurement of the condition?; Q5: Were confounding factors identified?; Q6: Were strategies to deal

with confounding factors stated?; Q7: Were the outcomes measured in a valid and reliable way?; Q8: Was appropriate statistical analysis used?).

*RCT (Q1: Was true randomization used for assignment of participants to treatment groups?; Q2: Was allocation to treatment groups concealed?; Q3: Were treatment groups similar at the baseline?; Q4: Were participants blind to treatment assignment?; Q5: Were those delivering treatment blind to treatment assignment?; Q6: Were outcomes assessors blind to treatment assignment?; Q7: Were treatment groups treated identically other than the intervention of interest?; Q8: Was follow up complete and if not, were differences between groups in terms of their follow up adequately described and analyzed?; Q9: Were participants analyzed in the groups to which they were randomized?; Q10: Were outcomes measured in the same way for treatment groups?; Q11: Were outcomes measured in a reliable way?; Q12: Was appropriate statistical analysis used?; Q13: Was the trial design appropriate, and any deviations from the standard RCT design (individual randomization, parallel groups) accounted for in the conduct and analysis of the trial?).
